# Malondialdehyde and TNF-α lowering effects of purified gambier (*Uncaria gambir* Roxb.) in diabetic rats

**DOI:** 10.1016/j.jaim.2023.100855

**Published:** 2024-01-23

**Authors:** Armenia Armenia, Elsa Badriyya, Sri Rahmita, Fitri Rachmaini, Rahmad Abdillah

**Affiliations:** a Departement of Pharmacology & Clinical Pharmacy, Faculty of Pharmacy, Universitas Andalas, Padang, West Sumatera, Indonesia; b Undergraduate Study Program, Faculty of Pharmacy, Universitas Andalas, Padang, West Sumatera, Indonesia

**Keywords:** Purified gambier, Catehin, Diabetes, Oxidative stress, MDA, TNF-α

## Abstract

Malondialdehyde (MDA) is one of a dominat marker in oxidative stress condition, and when inflammation occurred tumor necrosis factor- α (TNF-α**)** played a significant influence in the propagation this process. Purified gambier (*Uncaria gambier* Roxb.) contained 90% catechin which is proven to have antioxidant activity and may prevent unwanted inflammatory responses during diabetic state. **Objective:** The objective of this research was to assess how purified gambier affected plasma MDA and TNF- α levels in alloxan-induced diabetic rats. **Material and methods:** In this study, 35 rats were used. Alloxan 120 mg/kg BW intraperitoneal injection was administered to induce diabetes conditions in rats. All animals were divided into 5 groups, diabetic control group treated with vehicle, positive control group treated with glibenclamide dose 0.45 mg/kg BW), and treatment groups treated with purified gambier dose of 2.5; 5 and 10 mg/kg BW. All animals were treated respectively for 14 days. Plasma MDA and TNF- α levels were measured on day 3, and 14. **Results:** Two-way ANOVA was applied to analyze all of the data, these findings suggested that purified gambier has antioxidant-related anti-inflammation actions. possesses blood sugar-lowering activity (p<0.05). The plasma MDA and TNF- α level of treatment group were significantly reduced (p<0.05) compared to diabetes control group. **Conclusion:** These results depicted that at doses of 2.5–10 mg/kg BW, purified gambier has antioxidant-associated anti-inflammation effects when given for 14 days on diabetic rat model by reducing plasma levels MDA and TNF-α.

## 1 Introduction

Diabetes mellitus is growing rapidly all over the world. According to the International Diabetes Federation (IDF), the overall number of diabetic patients in Indonesia in 2019 was 10,7 million, with a projected growth to 13,7 million in 2030 and 16.9 million in 2045 [1]. This metabolic disease needs a long life treatment and thus costly. Almost none of the anti-diabetic drugs without side effect. Therefore, a new alternative anti-diabetic with more accomodated price and less side effect is needed [2].

Chronic hyperglycemia in diabetics results in an increase of reactive oxygen species (ROS). This internal mechanism triggers a chain reaction of oxydative stress [3]*.* Numerous antioxidant enzymes, including superoxide dismutase (SOD), catalase (CAT), glutathione peroxidase (GPx), and peroxiredoxin (Prx), would be inhibited in this circumstance [4].

ROS will interact with polyunsaturated fatty acids (PUFAs) of the lipid bilayers of the cell membrane, which produce lipid peroxidation. The latter produces the highly reactive aldehyde malondialdehyde (MDA) as a by-product, as well as other hazardous aldehydes. MDA served as the primary biomarker of oxidative stress [5]. Furthermore, oxidative stress in adipose tissue produces the adipocytokine TNF-, a pro-inflammatory mediator involved in insulin resistance [6]. That is why MDA and TNF-α levels in the specific condition of diabetes will be higher. Therefore, as a result, traditional medicinal plants with antioxidant properties are predicted to diminish oxidative stress and lower the amount of MDA and TNF-.α [7].

Gambier (*Uncaria gambier* Roxb.) is one of the traditional plants that found in Indonesia, esspecially in certain regions in Sumatera, such as West Sumatera, South Sumatera and Bengkulu. Gambier contains several active metabolic compounds, such as catechin (7-33%), catechu tannin acid (20-55), pyrocatechol (20-30%) and resin (1-2%) [8,9]. Indonesian people traditionally chewed gambier with dried areca nut and betel leaves. Furthermore, gambier leaves are used in a herbal treatment mixture to cure dysentery, diarrhea, deafness, spongy gums, and sore throat [10]. Gambier is also listed as a medicinal plant in the Indonesian Medicinal Pharmacopoeia monographs.

Several research have revealed that gambier possesses a variety of pharmacological properties, such as antioxidant [11], antibacterial [12], anti-inflammatory [13], anticancer [14], and xanthine oxidase inhibitor [15]. Other study by Arundita et al., in 2020 showed that purified gambier have effect alpha-glucosidase inhibitor activity with a better potency compared to acarbose [16]. Furthermore, our previous study also confirm that purrifed gambier have blood sugar and blood pressure–lowering activity [9]. However, there is limited information regarding its effect on reducing MDA and TNF-α in diabetic individuals. The purpose of this study is to investigate how dose and duration of administration of purified gambier affect MDA and TNF- plasma levels in diabetic rats.

## 2 Materials and methods

### 2.1 Plant materials

Andalas Sitawa Fitolab Ltd. Padang, West Sumatera, Indonesia provided purified gambier in compliance with Indonesian Herbal Pharmacopoea requirements.

### 2.2 Chemicals

Merck provided alloxan and glibenclamide (Germany), thiobarbituric acid, trichloroasetic acid, and standard malondialdehyde were obtained from Sigma Aldrich (USA) while Rat TNF-α ELISA Kit was obtained from BT Lab®, Shanghai, China.

### 2.3 Experiment animals

Male Wistar Kyoto rats weighing 180-220 kg were used in this research. Animals for standard laboratory usage were obtained from the Animal House Faculty of Pharmacy, Universitas Andalas. All studies were carried out in accordance with the European Council Directive on the Laboratory Animal Care and Use Animals (86/609/EEC). The protocol was authorized by the Faculty of Medicine's Research and Ethics Committee (No 604/UN.16.2/KEP-FK/2022).

### 2.4 Diabetic animal preparation

Alloxan of 120 mg/kg BW was injected intraperitoneal to the rat after 12 h starved. To avoid hypoglycemia, the animals were given a 10% glucose solution for three days following the alloxan injection. The fasting animal blood glucose was measured using *Easy Touch ® GCU.*

### 2.5 Experimental design

35 diabetic rats were divided into five groups of six rats each at random. Group I as the diabetic control, given vehicle (5% Na CMC). Group II was given glibenclamide at a dosage of 0.45 mg/kg BW as a positive control (Glibenclamide) Group III received purified gambier at a dosage of 2.5 mg/kg BW (PG 2.5). Group IV received purified gambier at a dosage of 5 mg/kg BW (PG 5). Group V received purified gambier at a dosage of 10 mg/kg BW (PG 10). All treatment regimens began 8 days after diabetes induction and were continued on a daily basis for 14 days.

### 2.6 Blood glucose measurement

On days 1, 3, 7, and 14, fasting blood sugar levels were monitored. An accu-check active glucometer was used to test blood glucose levels in the rat tail (Roche, Germany). The percentage of fasting blood glucose change was calculated using the equation as seen in our previous research [9,17].

### 2.7 Serum malondialdehyde measurement

Bir et al. explain the blood malondialdehide analysis using Thiobarbituric Acid Reactive Substance (TBARS) using spectroscopic analytical techniques [18].

### 2.8 Serum TNF-α measurement

TNF-alpha levels were measured in blood samples using the enzyme linked immunosorbent assays (ELISA) method with ELISA Kit (BT Lab®, Shanghai, China) and an ELISA microplate reader, as described in the manufacturer's protocol.

### 2.9 Data analysis

The data was analyzed using two-way ANOVA, followed by the Duncan Multiple Range Test (DMRT). To evaluate significance, the 95% confidence interval was employed.

## 3 Results

### 3.1 Alloxan induced diabetes

Alloxan induction in WKY rats resulted in diabetic pathology, as shown in Table 1. Fasting blood sugar levels exceeded 126 mg/dL 14 days after induction, as shown in Table 1. When compared to the period before induction, the animal test demonstrated clinical signs that confirmed the examination results, such as excessive urination, polyphagia, polydipsia, and altered behavior. Alloxan monohydrate 120 mg/kg BW caused hyperglycemia in WKY rats in this research.

**Table 1:** Effect of alloxan in experimental groups

Treatment	Parameters			
	SBP (mmHg)	DBP (mmHg)	HR (bpm)	BG (mg/dL)
Baseline	112.2 ± 3.4	79.0 ± 3.0	323.4 ± 7.2	88.7 ± 2.9
Induction	162.9 ± 2.7*	128.5 ± 2.5*	384.7 ± 6.4*	324.7 ± 5.8*

### 3.2 Effect of purified gambier on blood glucose

**Fig. 1:**
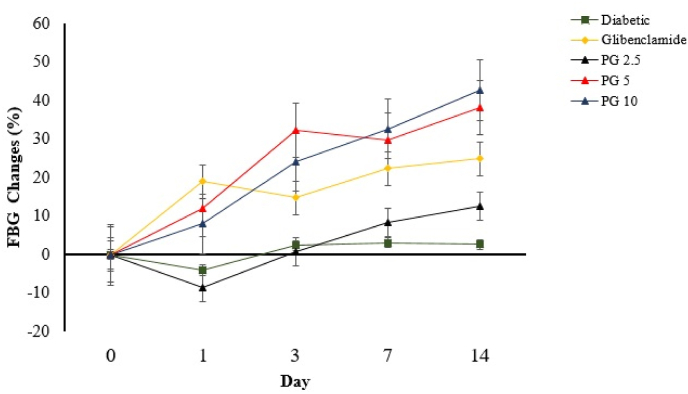
Effect of purified gambir on fasting blood glucose in diabetic rats. Each value is a mean of seven determinations.

PG considerably decreases fasting blood glucose levels (p<0.05), as shown in Fig. 1, the length of administration and the combination of treatment and time had no significant impact (p>0.05). When compared to the other groups, PG 10 had the greatest effect in lowering fasting blood glucose. Effect of purified gambier (*Uncaria* *gambier* Roxb.) on malondialdehyde plasma.

### 3.3 Effect of purified gambier (*Uncariagambier* Roxb.) on MDA

**Fig. 2:**
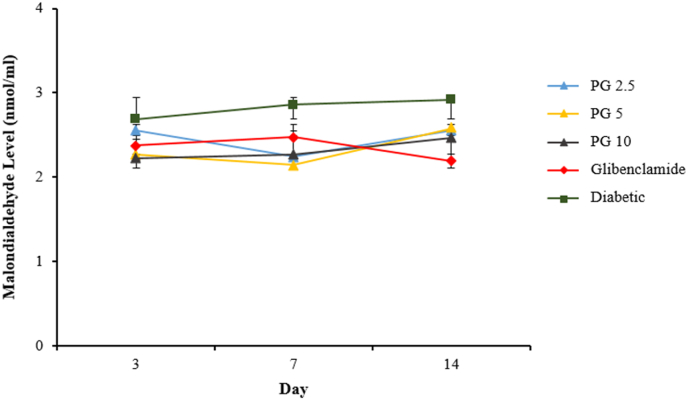
Effect of purified gambier on serum malondialdehyde levels diabetic rats. Each value is a mean of seven determinations ± SEM.

Purified gambier had a significant effect on decreasing serum malondialdehyde levels in diabetic rats (p<0.05), notwithstanding the fact that the period of administration had no impact (p>0.05) on the reduction in serum MDA levels in diabetic animals. Fig. 2 shows that there was no differences in the response of serum MDA levels across the PG groups. These results indicate that administering PG for 14 days in a diabetic animal was successful in lowering blood malondialdehyde concentrations.

### 3.4 Effect of purified gambier (*Uncariagambier* Roxb.) on TNF-α

**Fig. 3:**
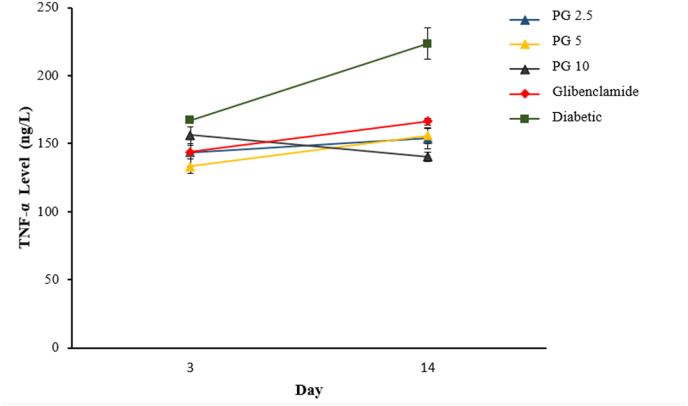
Effect of purified gambier on TNF-α Level in diabetic rats. Each value is a mean of seven determinations ± SEM.

Purified gambier had a significant effect on TNF-α levels in diabetic rats (p<0.05). However, there was no difference in the response of TNF-α level for all PG groups (p>0.05) as seen in Fig. 3. This study indicated that feeding PG for 14 days in a diabetic model can suppress TNF-α levels.

## 4 Discussion

The purpose of this study was to study the effects of purified gambier (*Uncaria gambier* Roxb) on the inflammatory and antioxidant stress produced by diabetic Mellitus (DM) in a rat model with insulin resistance and insufficiency. Several models have been developed to assess diabetes pathogenesis in experimental animals. In order to mimic varied pathological situations of actual diabetes for in vivo inquiry, the adoption of multiple models in evaluating the diabetes-related stress oxidative of a test medicine or plant extract is necessary. This research is crucial because it guarantees that ethnomedicinal implementations of PG are always supplemented by empirical pre-clinical evidence, significantly enhancing clinical value and commercialization. Diabetic rats in this research showed typical DM symptoms such as increased thirst, urination, and appetite.

As a diabetogen, alloxan selectively damages pancreatic beta-cell granules, causing insulin insufficiency and hyperglycemia. Alloxan promotes the emergence of reactive oxygen species (ROS). Alloxan will be reduced to dialuric acid, and then a process of reoxidation of dialuric acid will occur to alloxan again and produce ROS [19]. Our previous research found that after alloxan-induced diabetes, blood sugar levels can reach above 600 mg/dL, despite the fact that the alloxan dosage was significantly lower than that documented by Ighodaro et al. [20].

Glibenclamide was utilized as the control drug in this study. Glibenclamide is a sulfonylurea that increases beta-pancreatic activity and thereby increases insulin production. Glibenclamide was shown to be the most effective in producing hypoglycemic effects in alloxan-induced diabetes [21,22].

Diabetic subjects show altered levels of all oxidative stress biomarkers. Diabetes mellitus causes enhanced lipid peroxidation and peroxyl radical production due to hyperglycemia and dyslipidemia. Several investigations were conducted to assess the levels of stress-related biomarkers in both type 1 and type 2 diabetes [23]. In type 2 diabetes, elevated plasma and urine isoprostane levels indicated lipid peroxidation. MDA levels were also increased than in normal patients and linked with the extent of glycemic control achieved [24]. MDA causes collagen cross-linking and further covalent changes, causing a loss of elasticity and disruption in tissue remodeling, all of which contribute to the development of pathology within the organism, focusing on the blood vessel system [25].

Diabetes is characterized by persistent hyperglycemia and the development of micro and macrovascular complications. A gradual degenerative process triggers microvascular complications at the retinal, renal glomerulus, and peripheral nerve levels. Macrovascular disorders are characterized by the rapid formation of arteriosclerosis in the arteries supplying the lower limbs, brain and heart [21]. Long-term vascular involvement remain the leading cause of morbidity and mortality among diabetics [26]. Evidence from clinical and experimental studies indicates that oxidative stress is essential in the pathophysiology of diabetes-related disorders [27].

ROS accumulation will induce pancreatic beta cell damage and death, causing hyperglycemia. Hyperglycemia condition will continuously increase ROS production through autoxidation and glucose metabolism. Increasing ROS production will cause the accumulation of free radicals resulting in oxidative stress. When the number of free radicals in the body is higher than the number of antioxidants, it will induce cell apoptosis [28]. Many kinds of cells may be affected by this condition, such as endothelial tissue of blood vessels, kidneys and others. These all lead to diabetic complications [3].

Malondialdehyde (MDA) is a major biomarker of oxidative stress. This highly reactive aldehyde compound is produced by interacting ROS with polyunsaturated fatty acids (PUFAs) in the cell membrane's lipid bilayer. MDA levels in diabetic rats were found to be higher (Fig. 2), indicating an increased state of cellular oxidative damage. PEG administration dramatically decreased the generation of this ROS-induced peroxidation in diabetic conditions, reducing oxidative damage to these organs [29].

There is compelling proof that inflammatory processes, which are a constant companion of oxidative stress, play a significant role in the etiology of diabetes and its complications [30]. Despite from a decrease in the antioxidant defense system in diabetic rats, the current study revealed a considerable increase in TNF- plasma levels in diabetic animals (Fig. 3) [31].

TNF- α is a pro-inflammatory cytokine that has been linked with the pathophysiology of autoimmune disease and other inflammatory illnesses. Acute local TNF- α release generates a local inflammatory response to control infections. However, acute systemic TNF- release causes sepsis and shock. Elevated TNF- α is also harmful to glucose metabolism [32]. TNF- α can affect insulin sensitivity in a variety of ways, including by inhibiting insulin receptor signal transduction, lowering glucose transporter-4 in adipocytes, and suppressing adiponectin [33].

TNF-α causes renal damage by increasing epithelial cells permeable, altering intra-glomerular cerebral perfusion, causing invasive cells to migrate into the kidney, and activating apoptotic cell death [34]. TNF- α also promotes the production of genes producing IL-6 and MCP-1, which contributes to the advancement of atherosclerosis [27].

The results of this research show that pure gambier reduces fasting blood glucose levels as well as MDA and TNF- levels significantly compared to the diabetic group. What is concerning is that gambier had a substantially different effect on fasting blood glucose levels at a dose of 5 mg/kg BW. This impact, however, did not apply to MDA and TNF- levels. This phenomenon implies that a rise in dose wasn't proportional to an increase in efficacy in decreasing blood glucose levels. This effect is linked to the use of excessive doses of antioxidants [14,35].

As mentioned earlier, catechins are polyphenolic flavonoid compounds. Catechins are able to suppress oxidative stress by donating one electron from its hydroxy phenol group to free radicals to produce stable compounds. It may also inhibit pro-oxidant enzymes such as COX (cyclooxygenase) and iNOS (inducible nitric oxide synthase) [36]. These two enzymes are active in producing ROS. It is expected that the catechin of gambier reduces the number of ROS, which in turn prevent lipid peroxidation, cell and tissue damage, and inflammatory processes [15]. It could be understood why the diabetic animals treated with purified gambier showed a decrease in serum MDA and TNF-α levels in this study. By reducing MDA and TNF-α, the worsening of pancreatic cell damage and insulin resistance can be decelerated, and the disease progression and its complication will be slowed down [37,38].

Antioxidant supplementation has been advised due to its pharmacological properties in treating diseases. The use of antioxidants for high blood glucose has long been recommended, particularly for treatment options. This method, however, remains extremely hard due to inconsistent outcomes. Furthermore, disease improvement is rarely found, particularly in diabetic complications. A thorough evaluation of antioxidant supplementation is required, particularly for primary or secondary disease prevention.

## 5 Conclusion

Purified gambier reduced fasting blood glucose levels and, at the same time decreased MDA and TNF-α level in the alloxan-induced diabetic rats. This study suggests that administration of purified gambier at a dose of 2.5 mg/kg BW -10 mg/kg BW for 14 days in diabetic rats can possibly reduce MDA concentration and suppress TNF-α formation.

## Credit author statement

AA: Conceptualization, Methodology, Supervision, Funding acquisition; RA: Data curation, Writing-Original draft preparation, Writing - Review & Editing, Project administration; FR: Validation, Visualization, Investigation; SR: Investigation, Resources; EB: Formal analysis, Software, Validation.

## Funding

This study was supported by the Faculty of Pharmacy, 10.13039/501100014563Universitas Andalas, Indonesia (Grant number: 09/UN16.10.D/PJ.01/2022).

## Declaration of competing interest

The authors state that they have no conflicts of interest.
